# Supplementary material for: Assessing the use of prescription drugs and dietary supplements in obese respondents in the National Health and Nutrition Examination Survey
Source: PLoS One. 2022 Jun 3;17(6):e0269241. doi: 10.1371/journal.pone.0269241 (PMC9165812; doi:10.1371/journal.pone.0269241)
Supplement: S1 Table — (PDF) [file pone.0269241.s001.pdf]

S1 Table. NHANES Variables

NHANES File	NHANES Variable Name	NHANES Question	Reference in the Paper
Body Measures	BMXBMI	Body mass index	BMI
Demographic Variables and Weights	SEQN	Respondent sequence number	None
Demographic Variables and Weights	RIAGENDR	Gender	Sex
Demographic Variables and Weights	RIAAGEYR	Age in years at screening	Age
Demographic Variables and Weights	RIDRETH1	Race/Hispanic Origin	Race
Demographic Variables and Weights	WTINIT2YR	Full sample 2 year interview weight	None
Demographic Variables and Weights	INDHHIN2	Total household income	Annual household income
Dietary Supplement Use 30-Day - Individual Dietary Supplements	DSDCOUNT	Total # of Dietary Supplements Taken? (Includes all supplements and the antacids reported with supplements, but not antacids reported with medications.)	DS use
Health Insurance	HIQ011	Covered by health insurance?	Health Insurance
Income	INDFMMPI	Family monthly poverty level index (Family monthly poverty level index, a ratio of monthly family income to the HHS poverty guidelines specific to family size.)	PIR
Prescription Medications	RXDUSE	Taken prescription medication, past month	RXD use
Prescription Medications	RXDCOUNT	Number of prescriptions medications taken	Number RXD
Prescription Medications - Drug Information	RXDDCN1A	Drug category name - CAT 1, LEV 1	RXD category
