# Supplementary material for: Assessing the use of prescription drugs and dietary supplements in obese respondents in the National Health and Nutrition Examination Survey
Source: PLoS One. 2022 Jun 3;17(6):e0269241. doi: 10.1371/journal.pone.0269241 (PMC9165812; doi:10.1371/journal.pone.0269241)
Supplement: S2 Table — (PDF) [file pone.0269241.s002.pdf]

S2 Table. Reported prescription drug use by demographic characteristics among obese and control group.

Variable	Control				Obese			
	Odds ratio	95% Wald CL		P value	Odds ratio	95% Wald CL		P value
**Gender**								
Male	0.53	0.498	0.564	<.0001	0.569	0.52	0.623	<.0001
Female (reference)	1				1			
**Age Group**								
18-24	0.104	0.081	0.134	<.0001	0.076	0.049	0.119	<.0001
25-34	0.127	0.099	0.163	<.0001	0.115	0.075	0.178	<.0001
35-44	0.164	0.128	0.211	<.0001	0.225	0.146	0.346	<.0001
45-54	0.279	0.218	0.357	<.0001	0.404	0.262	0.622	<.0001
55-64	0.509	0.396	0.654	<.0001	0.746	0.482	1.154	0.1881
65-74	0.694	0.551	0.873	0.0019	0.839	0.54	1.302	0.4332
75 over (reference)	1				1			
**Race**								
Mexican American	0.468	0.411	0.533	<.0001	0.408	0.351	0.476	<.0001
Other Hispanic	0.592	0.51	0.688	<.0001	0.451	0.369	0.551	<.0001
Non-Hispanic White (reference)	1				1			
Non-Hispanic Black	0.607	0.544	0.677	<.0001	0.621	0.548	0.705	<.0001
Other Race – Including Multi-Racial	0.521	0.464	0.586	<.0001	0.64	0.514	0.796	<.0001
**PIR**								
0-1 (reference)	1				1			
1-2	0.962	0.87	1.064	0.4506	0.813	0.71	0.931	0.0028
2-3	1.026	0.917	1.148	0.6549	0.858	0.735	1.001	0.0518
3-4	0.953	0.847	1.073	0.4274	0.85	0.718	1.006	0.0583
4-5	1.095	0.962	1.246	0.1697	0.774	0.644	0.931	0.0066
>=5	1.201	1.078	1.338	0.0009	0.79	0.672	0.93	0.0046
**Covered by any insurance**								
Yes	1.597	1.305	1.955	<.0001	1.48	1.096	1.998	0.0105
No (reference)	1				1			
**Covered by private insurance**								
Yes	1.289	1.068	1.556	0.0082	1.603	1.206	2.129	0.0011
No (reference)	1				1			
**Covered by Medicare**								
Yes	2.424	1.967	2.986	<.0001	3.657	2.699	4.956	<.0001
No (reference)	1				1			
**Covered by Medicaid**								
Yes	1.93	1.546	2.409	<.0001	2.327	1.7	3.184	<.0001
No (reference)	1				1			
**Covered by other government insurance**								
Yes	1.829	1.487	2.251	<.0001	2.173	1.612	2.93	<.0001
No (reference)					1			
