# Supplementary material for: Assessing the use of prescription drugs and dietary supplements in obese respondents in the National Health and Nutrition Examination Survey
Source: PLoS One. 2022 Jun 3;17(6):e0269241. doi: 10.1371/journal.pone.0269241 (PMC9165812; doi:10.1371/journal.pone.0269241)
Supplement: S3 Table — (PDF) [file pone.0269241.s003.pdf]

S3 Table. Reported dietary supplements use by demographic characteristics among obese and control group.

Variable	Control				Obese			
	Odds ratio	95% Wald CL		P value	Odds ratio	95% Wald CL		P value
**Gender**								
Male	0.577	0.544	0.612	<.0001	0.582	0.537	0.63	<.0001
Female (reference)	1				1			
**Age Group**								
18-24	0.216	0.187	0.25	<.0001	0.274	0.208	0.361	<.0001
25-34	0.306	0.266	0.353	<.0001	0.333	0.257	0.431	<.0001
35-44	0.361	0.314	0.416	<.0001	0.363	0.283	0.467	<.0001
45-54	0.461	0.4	0.53	<.0001	0.537	0.42	0.688	<.0001
55-64	0.68	0.587	0.788	<.0001	0.832	0.651	1.065	0.1442
65-74	0.845	0.722	0.989	0.0361	0.924	0.741	1.153	0.4841
75 over (reference)	1				1			
**Race**								
Mexican American	0.57	0.506	0.641	<.0001	0.617	0.534	0.712	<.0001
Other Hispanic	0.7	0.609	0.804	<.0001	0.86	0.716	1.032	0.1058
Non-Hispanic White (reference)	1				1			
Non-Hispanic Black	0.537	0.484	0.596	<.0001	0.666	0.594	0.747	<.0001
Other Race – Including Multi-Racial	0.847	0.76	0.945	0.0028	1.077	0.882	1.315	0.4667
**PIR**								
0-1 (reference)	1							
1-2	1.036	0.945	1.136	0.4527	1.238	1.098	1.396	0.0005
2-3	1.109	1	1.23	0.0506	1.302	1.137	1.491	0.0001
3-4	1.238	1.109	1.383	0.0002	1.505	1.299	1.743	<.0001
4-5	1.36	1.204	1.537	<.0001	1.835	1.561	2.157	<.0001
>=5	1.715	1.549	1.9	<.0001	1.758	1.524	2.028	<.0001
**Covered by any insurance**								
Yes	1.411	1.255	1.587	<.0001				
No (reference)	1							
**Covered by private insurance**								
Yes	1.217	1.1	1.347	0.0002	1.332	1.212	1.464	<.0001
No (reference)	1				1			
**Covered by Medicare**								
Yes					1.335	1.12	1.591	0.0012
No (reference)					1			
**Covered by Medicaid**								
Yes	0.695	0.592	0.814	<.0001				
No (reference)	1							
**Covered by other government insurance**								
Yes					1.402	1.204	1.632	<.0001
No (reference)					1			
