# Supplementary material for: Assessing the use of prescription drugs and dietary supplements in obese respondents in the National Health and Nutrition Examination Survey
Source: PLoS One. 2022 Jun 3;17(6):e0269241. doi: 10.1371/journal.pone.0269241 (PMC9165812; doi:10.1371/journal.pone.0269241)
Supplement: S4 Table — (PDF) [file pone.0269241.s004.pdf]

S4 Table. Performance of machine learning models for classifying DS use

Model	With only demographic variables as predictors					After adding “RXD use” as a predictor				
	**Accuracy**	**Precision**	**Recall**	**F1**	**AUROC**	**Accuracy**	**Precision**	**Recall**	**F1**	**AUROC**
**Logistic Regression**	**0.646**	**0.633**	**0.604**	**0.62**	**0.7**	**0.651**	**0.636**	**0.614**	**0.625**	**0.703**
Naïve Bayes	0.644	0.621	0.641	0.63	0.698	0.641	0.615	0.647	0.631	0.699
Random Forest	0.631	0.612	,602	,607	0.67	0.624	0.602	0.609	0.606	0.664
SMO (SVM)	0.613	0.617	0.482	0.54	0.606	0.646	0.628	0.622	0.925	0.645
