# Supplementary material for: Assessing the use of prescription drugs and dietary supplements in obese respondents in the National Health and Nutrition Examination Survey
Source: PLoS One. 2022 Jun 3;17(6):e0269241. doi: 10.1371/journal.pone.0269241 (PMC9165812; doi:10.1371/journal.pone.0269241)
Supplement: S5 Table — (PDF) [file pone.0269241.s005.pdf]

S5 Table. Performance of machine learning models for classifying RXD use

Model	With only demographic variables as predictors					After adding "DS use" as a predictor				
	**Accuracy**	**Precision**	**Recall**	**F1**	**AUROC**	**Accuracy**	**Precision**	**Recall**	**F1**	**AUROC**
**Logistic Regression**	**0.743**	**0.77**	**0.753**	**0.76**	**0.816**	**0.743**	**0.768**	**0.758**	**0.763**	**0.818**
Naïve Bayes	0.739	0.759	0.765	0.76	0.813	0.738	0.757	0.768	0.762	0.813
Random Forest	0.73	0.745	0.768	0.76	0.799	0.724	0.742	0.757	0.75	0.791
SMO (SVM)	0.716	0.749	0.722	0.74	0.716	0.716	0.68	0.71	0.694	0.716
