# Supplementary material for: Assessing the use of prescription drugs and dietary supplements in obese respondents in the National Health and Nutrition Examination Survey
Source: PLoS One. 2022 Jun 3;17(6):e0269241. doi: 10.1371/journal.pone.0269241 (PMC9165812; doi:10.1371/journal.pone.0269241)
Supplement: S7 Table — (PDF) [file pone.0269241.s007.pdf]

S7 Table. Performance of machine learning models for classifying RXD use using PIR

**Model**	With only demographic variables as predictors					After adding "DS use" as a predictor				
	**Accuracy**	**Precision**	**Recall**	**F1**	**AUROC**	**Accuracy**	**Precision**	**Recall**	**F1**	**AUROC**
**Logistic Regression**	**0.743**	**0.772**	**0.75**	**0.76**	**0.816**	**0.743**	**0.769**	**0.756**	**0.762**	**0.818**
Naïve Bayes	0.737	0.756	0.766	0.76	0.81	0.736	0.755	0.763	0.759	0.809
Random Forest	0.736	0.755	0.764	0.76	0.809	0.734	0.75	0.769	0.759	0.803
SMO (SVM)	0.716	0.749	0.722	0.74	0.716	0.716	0.749	0.722	0.735	0.716
