# Supplementary material for: Assessing the use of prescription drugs and dietary supplements in obese respondents in the National Health and Nutrition Examination Survey
Source: PLoS One. 2022 Jun 3;17(6):e0269241. doi: 10.1371/journal.pone.0269241 (PMC9165812; doi:10.1371/journal.pone.0269241)
Supplement: S8 Table — (PDF) [file pone.0269241.s008.pdf]

Table S8. Performance of machine learning models for classifying RXD use into categories

Model	Classifying into drug count groups				
	**Accuracy**	**Precision**	**Recall**	**F1**	**AUROC**
**Logistic Regression**	**0.533**	**0.491**	**0.533**	**0.492**	**0.76**
Naïve Bayes	0.527	0.496	0.527	0.501	0.755
Random Forest	0.501	0.473	0.501	0.482	0.723
SMO (SVM)	0.509	--	0.509		0.657
