# Supplementary material for: Assessing the use of prescription drugs and dietary supplements in obese respondents in the National Health and Nutrition Examination Survey
Source: PLoS One. 2022 Jun 3;17(6):e0269241. doi: 10.1371/journal.pone.0269241 (PMC9165812; doi:10.1371/journal.pone.0269241)
Supplement: Attachment — Submitted filename: Response to Review_PLOSONE.docx [file pone.0269241.s010.docx]

**Response to Review**

Journal Requirements:

Please review your reference list to ensure that it is complete and correct. If you have cited papers that have been retracted, please include the rationale for doing so in the manuscript text, or remove these references and replace them with relevant current references. Any changes to the reference list should be mentioned in the rebuttal letter that accompanies your revised manuscript. If you need to cite a retracted article, indicate the article’s retracted status in the References list and also include a citation and full reference for the retraction notice.

***** Response: We have reviewed the reference list to make sure it is complete and correct.

**Additional Editor Comments:**

Dear authors, we appreciate your effort in addressing reviewers' comments. However, please directly respond to reviewer #2's comment regarding not adding newer NHANES 2015-2018 data in your manuscript. The current analysis using the 2003-2014 NHANES data is dated and might compromise implications of your findings.

***** Response: We have added the data of NHANES survey cycles 2015-2016, 2017-2018 in the revised version. We have re-done all the analyses and updated the results accordingly.

In addition, I suggest you to further revise the title and/or abstract to reflect the objective of the paper. Your objective was to examine the use of prescription drugs and dietary supplements by the individuals with obesity, but the current title and abstract (results section) only focus on use of prescription drugs. The aspect of dietary supplements is missing in title and abstract (results section). Please also double check the results in the abstract to reflect the most up to date data based on the revision. Thanks.

***** Response: We revised the title and abstract to reflect the objective of the paper.

**Reviewer #2**: The authors addressed almost all comments, except for comment 3 which was not fully addressed. The authors may need to update the analysis including the most recent data cycles (2015-2018) which are available now. In addition, the Figure 2C is hard to tell the statement "the largest maximum number of DS used is among people with BMI from 18-22 kg/m2", so this figure may need some modification and improvement.

***** Response: We have added the data of NHANES survey cycles 2015-2016, 2017-2018 in the revised version. We have re-done all the analyses and updated the results accordingly. For better clarity, we removed the two sentences “The largest maximum number of DS used (24) can be found among people with BMI from 18 to 22 kg/m^2^. The highest average number of RXD used can be found among people with BMI from 63 to 67 kg/m^2^.”
